# Fatty acid oxidation: driver of lymph node metastasis

**DOI:** 10.1186/s12935-021-02057-w

**Published:** 2021-07-03

**Authors:** Mao Li, Hong-chun Xian, Ya-Jie Tang, Xin-hua Liang, Ya-ling Tang

**Affiliations:** 1 State Key Laboratory of Oral Diseases and National Clinical Research Center for Oral Diseases, Department of Oral Pathology, West China Hospital of Stomatology, Sichuan University, Chengdu, China; 2 State Key Laboratory of Oral Diseases and National Clinical Research Center for Oral Diseases, Department of Oral and Maxillofacial Surgery, West China Hospital of Stomatology, Sichuan University, Chengdu, China; 3 State Key Laboratory of Microbial Technology, Shandong University, Qingdao, 266237 China

**Keywords:** Fatty acid oxidation, Lymph node metastasis, YAP, Lymph node pre-metastatic niche, Metabolic reprogramming, Immune suppression, Prognosis

## Abstract

Fatty acid oxidation (FAO) is the emerging hallmark of cancer metabolism because certain tumor cells preferentially utilize fatty acids for energy. Lymph node metastasis, the most common way of tumor metastasis, is much indispensable for grasping tumor progression, formulating therapy measure and evaluating tumor prognosis. There is a plethora of studies showing different ways how tumor cells metastasize to the lymph nodes, but the role of FAO in lymph node metastasis remains largely unknown. Here, we summarize recent findings and update the current understanding that FAO may enable lymph node metastasis formation. Afterward, it will open innovative possibilities to present a distinct therapy of targeting FAO, the metabolic rewiring of cancer to terminal cancer patients.

## Background

Metabolic reprogramming underwent by many types of cancer as well as diverse types of immune and stromal cells to fulfill the needs of uncontrolled growth and metastatic progression, is now tightly identified as a hallmark of cancer [1]. Normal cells mainly produce energy through oxidative phosphorylation, but tumor cells obtain energy through glycolysis even under normal oxygen concentration, which is a well-known Warburg effect [2], the first adaptive event in tumor metabolism. Since then, growing researches have been done to understand metabolic reprogramming in cancer. In addition to the Warburg effect, cancer cells can also rewire other metabolic ways, taking for example the upregulation of de novo lipid synthesis, fatty acid oxidation (FAO), amino acid oxidation and glutaminolysis [3]. These alterations are essential for supporting cancer cells growth and proliferation in unfavorable tumor microenvironment (TME) or metastatic sites, thereby targeting metabolism considered as relevant cancer therapy strategy. Accumulating evidence supports the presence of dynamic changes in the metabolism of metastasizing cells, contributing to their ability to successfully colonize in the distant organs. Now, with a greater depth of understanding FAO, it is well recognized that FAO plays an important role in proliferation, survival, stemness, drug resistance, and metastatic progression. Especially, it is further heightened that during metastasis, cancer cells are dependent on FAO in nutrient- and oxygen-depleted environmental conditions [4].

Malignant tumor is the leading cause of mortality, notably 90% of cancer patients dying of metastasis [5]. Lymph node metastasis (LNM), one of the most common ways of metastasis, is associated with progression, poorer prognosis and therapeutic schedule in numerous cancers, including breast [6], bladder [7] and gastric [8] cancer. In addition, lymph nodes of metastatic cancer are crucial sites for tumor-immune cells interaction and potential gateways for further dissemination of tumor cells to other metastatic sites [9, 10]. As the lymph flows into the systemic circulation through the thoracic duct or lymphovascular shunts, LNM can be identified as a side loop of the hematogenous metastasis circuit and interaction of chemokines, such as CXCR3, CXCR4, and CCR7 [11].

It has been shown that lymph node is the lipid-rich microenvironment where lymph node metastatic tumor cells may preferentially use fatty acids as energy source [12].A research in *Science* has discovered that FAO is the emerging factor of LNM [12], indicating that a time for the metabolic change enabling cancer cell to form LNM is coming. There are numerous studies indicating different ways how tumor cells metastasize to the lymph nodes, but the mechanism by which FAO contributes to LNM remains unclear. Investigation on the relationship between FAO and LNM can present an emerging picture on how FAO support LNM formation in order to help open innovative possibilities to treat cancer. Therefore, in this review, we give the presentation about a significant role which FAO plays in LNM and mechanism by which FAO provides motivation for LNM.

## Fatty acid β-oxidation, the major pathway of fatty acid decomposition

**Fig. 1:**
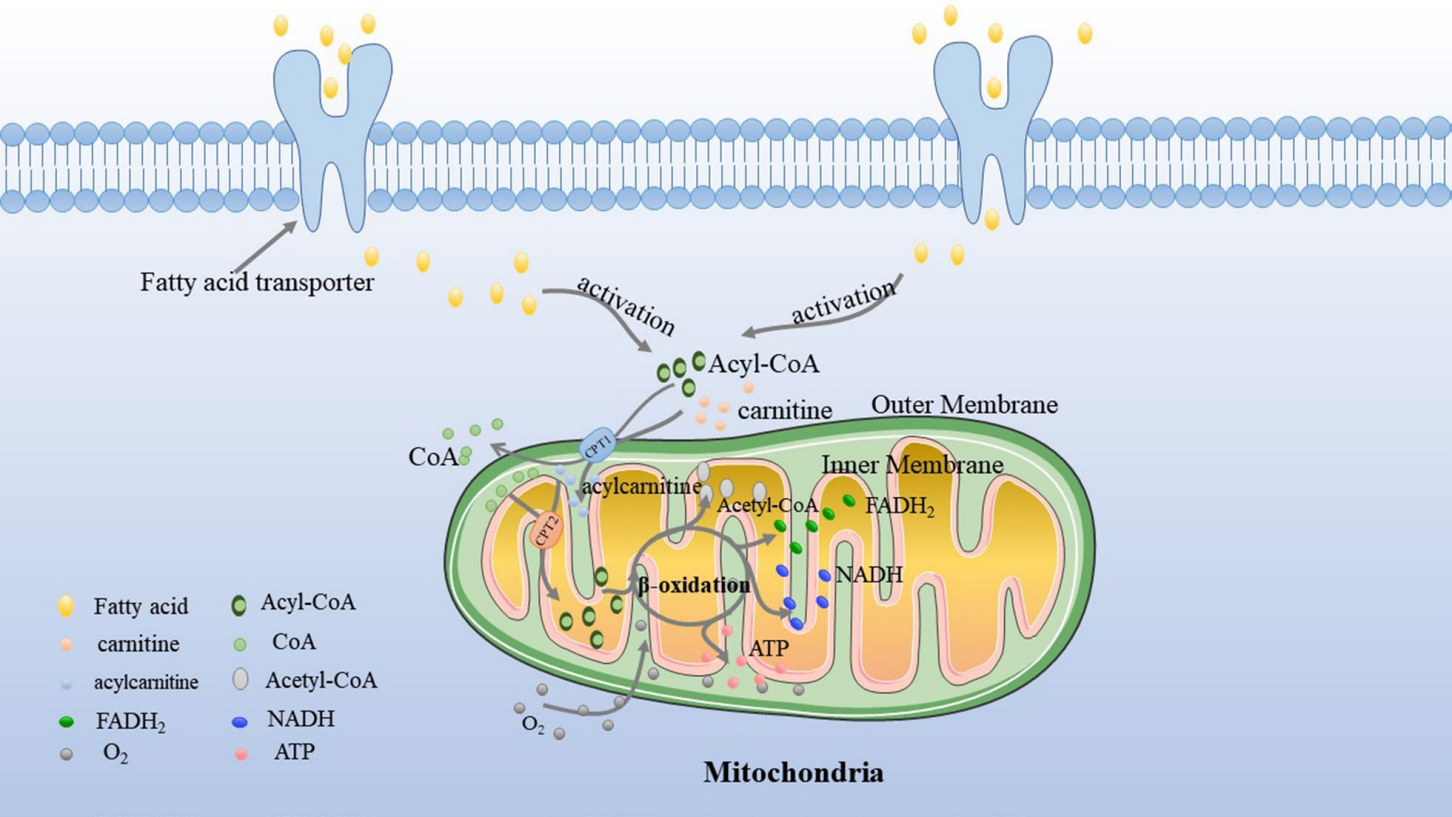
FAO Basics. After entering into the cell via fatty acid protein transporters, fatty acids are activated to acyl-CoA. The carnitine carnitine palmitoyltransferase system then transports acyl-CoA from cytoplasm into mitochondrial matrix for oxidation: CPT1 converts acyl-CoA into acylcarnitines and CPT2 converts acylcarnitine back into acyl-CoA for oxidation

Fatty acid β-oxidation, referred to as FAO, is the major pathway of fatty acid decomposition and a multi-step catabolic process, meaning that the fatty acid is converted into acetyl-CoA which will be fully oxidized to generate ATP to support living. Fatty acids transferred to cells is the first step of FAO via fatty acid protein transporters on the cell surface, e.g. fatty acid translocase (FAT/CD36), tissue specific fatty acid transport proteins (FATPs), and plasma membrane bound fatty acid binding proteins (FABPs) [13]. Because FAO occurs in the mitochondrial matrix, the fatty acid molecule has to be activated to form acyl-CoA by acyl CoA synthetase before entering into mitochondrion [14]. Long-chain acyl-CoA requires a special transport mechanism to cross the outer mitochondrial membrane. The process is that acyl-CoA forms fatty acylcarnitine at the outer mitochondrial by the action of carnitine palmitoyltransferase 1 (CPT1), the rate-limiting step for mitochondrial β-oxidation, and this is then transported to the intramembrane space [15]. Then, the fatty acylcarnitine transported into the inner mitochondrial membrane is through carnitine palmitoyltransferase 2 (CPT2) in order to release free carnitine and acyl-CoA. Finally, under the action of many enzymes, acyl-CoA is broken down to produce acetyl-CoA, FADH_2_ and NADH in the mitochondrial matrix. In cancer, FAO can promote the production of ATP when required, inhibit pro-apoptotic pathways and provide metabolic intermediates for cancer cell growth [16] (Fig. 1).

The most prominent transcriptional regulators of FAO are peroxisome proliferator-activated receptors (PPARs) of the ligand-activated nuclear receptor superfamily. There are three subtypes of the PPARs, PPARα, PPARγ and PPARδ. PPARα has been shown to stimulate CPT1A transcription in the liver by binding to the peroxisome proliferator response element on the CPT1A gene promoter. PPARδ activation has been shown to increase muscle FAO and to regulate several genes implicated in fatty acid uptake and metabolism in rodent models [17]. In addition, the FAO rate is tightly controlled by the activity of AMP-activated protein kinases (AMPKs). Activation of AMPK stimulates FAO primarily through phosphorylation and inactivation of its downstream target acetyl-CoA carboxylase (ACC) which possess two isoforms, ACC1 and ACC2.

## FAO promoting metastasis through substrates for FAO, fatty acid protein transporters, and FAO enzymes or their regulators

FAO has been shown to be pivotal to develop and maintain the malignant phenotype of cancer cells in unfavorable TME. Either genetically or pharmacologically inhibition of FAO reduces ATP supply and impairs cell proliferation in prostate cancer [18], bladder cancer [19] as well as diffuse large B-cell lymphoma [20] and malignant glioma cell [21]. Then, FAO, one of the altered tumor metabolism ways, has been further found to be closely associated with successful metastatic colonization. For example, the key FAO transcriptional regulator, peroxisome proliferator-activated receptor gamma coactivator 1 alpha (*PPARGC1A*, also known as PGC-1α) is highly expressed in triple negative breast cancer [22]. There is a research reporting that if breast tumor cells highly express the aldo–keto reductase *AKR1B10*, they will increase the utilization of FAO in distant organ compared with primary cancer [23]. It is notable that *AKR1B10* expression in human breast cancers positively correlated with *PPARGC1A* [23]. However, the molecular mechanism of *AKR1B10* promoting FAO in breast cancer metastasis via *PPARGC1A* remains unclear.

Fatty acids are the basement of FAO, which are important in building blocks of lipids in non-cancer and cancer cells membranes [24] and can serve as important signal molecules [25]. Acetate, a short-chain fatty acid, induces cancer cell apoptosis in colorectal cancer via mitochondrial changes, such as swelling and increased ROS production [26, 27] but facilitates tumor growth and metastasis in an acetyl-CoA synthetase 2- and hypoxia-inducible factor-2-dependent manner during low oxygen states or hypoxia in vivo [28]. Oleic acid, a monounsaturated fatty acid, can protect tumor cells from ferroptosis, an arduous challenge which hinders circulating tumor cells (CTCs) from hosting to distant organs by reducing the amount or the density of polyunsaturated fatty acids available for oxidation in membranes [29, 30]. This is why melanoma cells getting oleic acid protection from lymph nodes are more resistant to ferroptosis and easier to form distant metastasis through blood [31]. Fatty acids are required for cancer cells survival in the process of metastasis.

Fatty acid protein transporters are also associated with metastasis. CD36, a cell surface protein taking lipid up from the extracellular environment and modulating lipid metabolism, can be served as an effective marker to activate FAO [32]. Overexpression of CD36 in oral squamous cell carcinomas can be used to separate high metastasis-initiating potential tumor cells from multiple cell lines and patient-derived cells greatly increased their potential to metastasize to lymph nodes [33]. Additionally, it has been proved that high expression of FABP5 in positive connection with the presence of LNM of cervical cancers [34] can induce expression of matrix metalloproteinase-2 and matrix metalloproteinase-9 [35]. Importantly, FABP5, as a promising predictor of LNM, promotes metastasis by reprogramming fatty acids metabolism in cervical cancers [36].

Besides, enzymes involved in FAO contribute to metastasis. High expression of CPT1A in the models of epithelial-derived high-grade serous ovarian cancer cell dissemination contributes to resisting anoikis, a form of special apoptosis resulted from detachment from extracellular matrix and a significant role in metastasis [37]. Furthermore, anoikis drives a metabolic reprogramming towards FAO to maintain cellular energy demands [37] whereas glucose is used to keep the balance of redox [38]. A research uncovered that the expression of CPT1B up-regulated by phosphatidylinositol transfer protein, cytoplasmic 1 promotes omental metastasis of gastric cancer through metabolic rewiring—FAO [39].

The occurrence of FAO comprises a plurality of parts, fatty acids, fatty acid protein transporters and enzymes. If one of these is deficient, it will cause FAO disorders to block energy supply. That is, cancer metastasis may also be affected.

## FAO inducing epithelial to mesenchymal transition

**Fig. 2:**
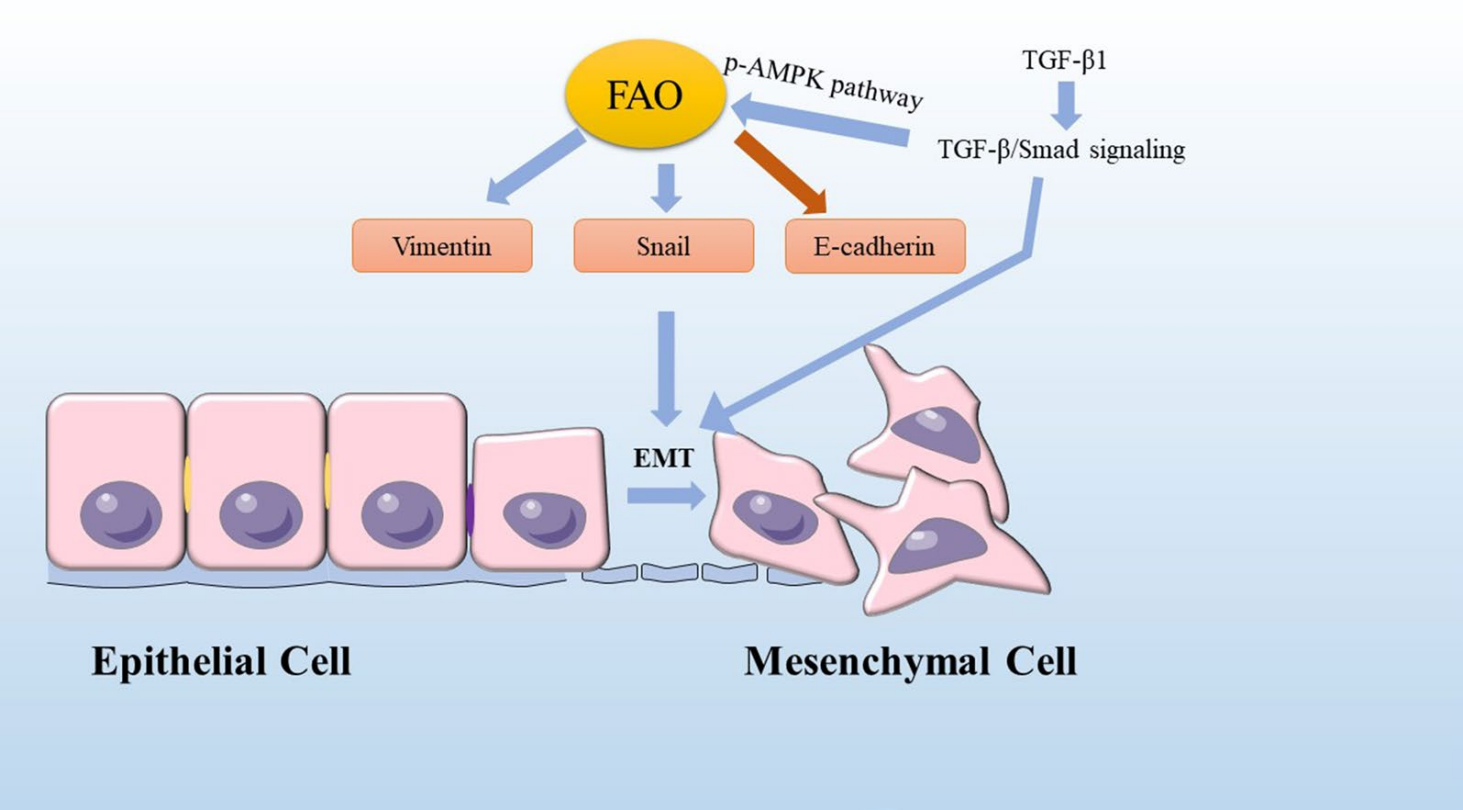
FAO and EMT. Some markers of EMT, such as Vimentin, Snail and E-cadherin are regulated by FAO. Vimentin and Snail are upregulated but E-cadherin is decreased, which display a start of cancer invasion. FAO is enhanced via the p-AMPK pathway owing to TGF-β1 expression activating TGF-β1/Smad signaling to boost EMT

Epithelial to mesenchymal transition **(**EMT) involving stemness, invasion, migration, apoptotic resistance, and metabolic reprogramming in cancers [40] is a reversible cellular program that transiently places epithelial cells into quasi-mesenchymal cell states [41]. EMT facilitates local invasion of cancer cells into adjacent tissues, which conduces to intravasation of cancer cells into blood or lymphatic vessels, a critical process of LNM. CPT1A overexpression promotes LNM via upregulating the expression of Vimentin, Snail and decreasing E-cadherin in gastric cancer, those markers of EMT [42]. Elevated the mitochondrial ROS level induced by FAO activates the p38 mitogen-activated protein kinase (MAPK) signaling pathway, resulting in the EMT of ROS-high tumor spheres cells, thereby potentiating caner invasion and metastasis in vitro [43]. EMT transcription factor Snail suppressing ACC2 leads to an increase in mitochondrial FAO, which allows pro-survival of breast cancer cells [44]. Importantly, combinatorial pharmacologic inhibition of pentose phosphate pathway and FAO with clinically available drugs efficiently reverts Snail-mediated metabolic reprogramming and suppresses metastatic progression of breast cancer cells [44]. It is reported that transcriptionally up-regulating TGF-β1 expression, giving rise to activating TGF-β1/Smad signaling to boost EMT can contribute to breast cancer invasion to lymph nodes [45], which increases FAO and OXPHOS activity via the p-AMPK pathway [46]. FAO induction in colon cancer cells by co-cultivation with adipocytes is connected with induction of EMT owing to decreased expression of E-cadherin and increased expression of Vimentin [47]. In prostate cancer (PCa), FABP12-PPARγ pathway activation has a novel role in metastasis through induction of EMT and lipid bioenergetics—the ATP production from FAO [48] (Fig. 2).

## YAP needed in FAO of LNM

A number of studies have shown that the abnormal regulation of the Hippo pathway will lead to the occurrence of cancer, so the key proteins of the Hippo pathway are expected to become therapeutic targets. YAP is a potent oncogene whose abnormal regulation can cause tumorigenesis. By using comparative transcriptomics and metabolomics analyses, a research in *Science* revealed that LNM requires tumor cells to undergo a metabolic shift toward FAO [12]. LN-metastatic tumors accumulate bile acids identified as potential molecular triggers to activate YAP mainly via vitamin D receptor, leading to FAO activation and successful adaptation to the LN microenvironment [12]. The FAO inhibitor etomoxir or depletion of YAP reduces LN metastasis in the melanoma mouse model, without affecting the growth of the primary tumor or the size of the LN, compared with untreated melanoma-bearing mice. However, the mechanisms by which YAP regulates FAO and by which FAO enables tumor growth in this context are unclear. Recently, it has been indicated that metabolic cues of lipids regulate YAP/TAZ activity [49]. Long-chain acyl-CoA dehydrogenase (ACADL) is a mitochondrial enzyme that is responsible for catalyzing the initial step for the β-oxidation of long-chain fatty acyl-CoAs. ACADL plays a tumor-suppressor role by inhibiting YAP activation in hepatocellular carcinoma, which is independent of its function in FAO [50]. Fatty acids identified as the substrate of FAO have a role in regulating YAP/TAZ, such as unsaturated fatty acids and palmitic acids. Palmitic acid induces expression of the Hippo pathway kinase MST1 and YAP phosphorylation, therefore inhibiting nuclear YAP and its function [51]. However, it is still unclear how YAP activation confers FAO, so further research will be needed.

## FAO associated with lymph node pre-metastatic niche

In 1989, Paget et al. [52] proposed the concept of “seed and soil”, describing that specific tumor cells (seeds) are more likely to transfer to specific organs (soil), and only when the soil is suitable for seeds will they grow and disseminate successfully. In 2005, Professor Kaplan first formulated the hypothesis of pre-metastatic niche, holding that a large number of cells induced by tumor cells and tumor-derived molecules enter the organ to be transferred before tumor cells, preparing soil for tumor transfer and indicating the direction of chemotaxis of tumor seeds into the organ [53]. The formation of lymph node pre-metastatic niche that is ready for metastasis is characterized by lymphangiogenesis, the recruitment of immunosuppressive cells, the up-regulation of chemokines and cytokines as well as vascular remodeling [54].

### Lymphangiogenesis

LNM is obviously connected with lymphangiogenesis–the formation of lymphatic vessels [55]. Primary tumors may motivate lymphangiogenesis to arrange for LNM and excess lymphangiogenesis favors metastasis and inflammation [56, 57]. A research revealed that a high density of tumor-associated macrophages (TAMs) significantly associated with pathologically positive lymph nodes may induce lymphangiogenesis and prepare an environment that facilitates cancer proliferation in LNs [58].

Lymphangiogenesis is a result of proliferation and differentiation of lymphatic endothelial cells which are differentiated from vein endothelial cells. This differentiation process is mainly achieved by direct binding of transcription factor PROX1 and induction of transcriptional expression of a variety of lymphatic genes [59]. However, a research in 2017 indicated that FAO is a metabolic regulator in lymphangiogenesis [60]. A study testified that FAO promotes nucleotide synthesis for supporting DNA replication as well as mediates epigenetic changes of histone acetylation, contributing to transcription of key lymphatic genes, and thereby furthering venous-to-lymphatic endothelial cell differentiation [61]. CPT1A, a rate-controlling enzyme in FAO, is indispensable for lymphangiogenesis. Wong et al. [60] unveiled that genetic and biochemical disruption of CPT1A function suppresses lymphangiogenesis. Although pharmacological blockade of CPT1 inhibits injury-induced lymphangiogenesis, lowering FAO seems to achieve complete inhibition of pathological lymphangiogenesis in cancer [62]. And it has been verified that lymphangiogenesis defects caused by FAO inhibition can be offset by acetate supplementation [60]. Acetyl-CoA generated by FAO subsequently enters into the Krebs cycle and nucleotide synthesis for proliferation important for epigenetic regulation associated with lymphangiogenesis [63]. Acetylation reactions of histone H3 at lysine 9 by p300 can use acetyl-CoA to regulate protein function [64], and acetylation of histones is of particular importance for epigenetic regulation of gene expression associated with lymphangiogenesis [63]. And FAO-derived acetyl-CoA supports PROX1-induced vascular endothelial growth factor receptor 3 (VEGFR3) expression which promotes lymphangiogenesis [60]. Shang and colleagues indicated that lymphangiogenesis is promoted by FABP5-mediated fatty acid metabolism in lymph node pre-metastatic niche through LNMICC (lncRNA associated with LNM in cervical cancer) [65].

### The recruitment of immunosuppressive cells

Some regulatory or immunosuppressive cells, such as regulatory CD4^+^ T cells (Tregs), myeloid-derived suppressor cells (MDSCs), TAMs, and tolerogenic dendritic cells (DCs) within the pre-metastatic niche potentially suppress anti-tumor immune responses. Evidence suggests that pre-metastatic niches provide microenvironment critical for cancer cell recruitment and survival to facilitate metastasis in non-small cell lung cancer [66], cutaneous squamous cell carcinoma [67] and breast cancer [68]. In line with this, the formation of advanced micro-metastasis also implies the formation of an immunosuppressive microenvironment in lymph nodes [69].

Cancer-associated immune cells make their metabolic pathways reprogrammed, which may contribute to immune suppression and tumor-promoting microenvironment [3]. T cells and effector T (T_eff)_ cells have been clearly shown to rely on glycolysis [70]. However, investigators have also revealed that when being at a disadvantage in the process of competing for glucose with tumor cells [71], T_eff_ cells change their metabolic mode into fatty acids energy supply via activation by STAT3 regulation, inhibiting anti-tumor response in obesity-promoted breast tumor [72, 73]. On the other hand, the energy metabolic switch of CD8^+^ T memory (T_mem_) cell can be orchestrated into FAO via tumor necrosis factor receptor-associated factor 6 to promote memory cell generation and protective immunity, which is different from the way that T cells use glucose as their main energy source [74]. In addition, the activation of FAO regulated by IL-15 promotes the development and maintenance of T_mem_ cells [75]. A latest paper revealed that tissue-resident T_mem_ cells infiltrated in the tumor microenvironment of gastric adenocarcinoma rely on FAO for cell survival [76]. Genetic and inhibitor data supported that germinal center B cells oxidize fatty acids to supply energy while conducting minimal glycolysis [77].

Foxp3 in Tregs may increase the protein level of acetyl CoA synthetase and CPT1A, which gives an excellent explanation that Tregs take in increasing fatty acids [78]. Investigation on PD-1 ligation found that PD-1 alters metabolic reprogramming of activated T cells which are unable to engage in glycolysis but are active and have an increased rate of FAO, thus influencing effector cells development [79]. The enhancement of FAO also can be detected in pre-activated CD4^+^ T cells accounting for the regulation of PD-1 blocking PI3K/Akt and MEK/Erk pathways which regulate glycolysis [79]. Blockade of FAO enhances antitumor immunity and improves the efficacy of anti-PD-1 inhibitors [80]. A study indicated that hypoxia-inducible factor 1α (HIF-1α), a metabolic switch, directs glucose away from mitochondria and leaves Tregs dependent on fatty acids for mitochondrial oxidation in glioblastoma, thereby enhancing Tregs immunosuppressive capabilities [81]. Michalek and his colleagues proposed that FAO inhibition also decreases Tregs function [82].

In contrast to glycolysis in M1 macrophages [83], FAO in M2 macrophages dominate [84, 85]. FAO plays a great role in not only M2 macrophages polarization [86], but also in IL-1β secretion of M2 macrophage, thus promoting tumor metastasis [87]. Macrophages without CPT2 have no ability to achieve FAO yet still seemed to fully polarize toward an M2 state after stimulation with IL-4, but treatment with etomoxir, an inhibitor of CPT1, potently blocks the M2 polarization of wild-type macrophages [88]. A significant body of researchers have been focused on the pharmacologic activity of etomoxir. But there are two key findings showing that etomoxir induces CPT1A-independent off-target effects in M2 macrophages and Tregs [89, 90]. Furthermore, by using genetic depletion of CPT1A, the Luciana Berod lab nicely demonstrated that CPT1A and FAO are not required for Tregs differentiation and function [90], which is quite amazing discovery.

It has been documented that MDSC regulated by periostin accumulates to develop immunosuppressive function during the early stage of breast tumor metastasis [68]. The energy metabolism pathway of MDSC is to activate FAO, so FAO inhibition blocks the immunosuppressive function of MDSC, thus allowing T cells to kill tumor cell [91]. Besides, there is an increase in fatty acids uptake and expression of FAO-related enzymes which can be found in human MDSC in peripheral blood and tumors [91]. It is revealed that myeloid cells undergo metabolic rewiring from glycolysis to FAO, a process that is accompanied by the stimulation of the immunosuppressive mechanisms, namely an increased production of arginase I, an increased expression of NOS2 and an increased production of ONOO−, which lead to enhancing the ability to suppress T cells response [92].

An important discovery showed that metabolic pathways modulate immunogenicity of murine DCs [93]. Through analyzing the underlying metabolic activity of DCs from immunogenic to tolerogenic stage, tolerogenic DCs, in contrast to mature DCs, display a significantly increased catabolic pathway related to FAO [94]. Inhibition of FAO restrains the function of tolerogenic DCs and partially restores T cells’ stimulatory capacity, demonstrating their dependence on this pathway [94].

### The up-regulation of chemokines and cytokines

In pathological conditions including cancer, not only lymphatic vessels but lymph nodes engage in disease progression and undergo remodeling in response to growth factors, chemokines and other signaling molecules secreted by tumor cells or other cells in pre-metastatic niches [62], including TGF-β, epidermal growth factor, vascular endothelial growth factor-C (VEGF-C) and CCR7 signaling and CCL1/CCR8. Recently, studies have shown that various chemokines and cytokines secreted by stromal cells and immune cells in pre-metastatic niches are associated with FAO. It has been shown that TGF-β is of importance in pelvic LNM in early-stage cervical cancer [95] and is a driver of apoptosis of DCs leading to fewer DCs in pre-metastatic lymph nodes than that of no metastasis in non-small cell lung cancer [96]. In gastric cancer, TGF-β1 secretion by mesenchymal stem cells augments the activation level of SMAD2/3 through TGF-β receptors, which then enhances expression of long non-coding RNA MACC1-AS1 and promotes FAO-dependent stemness and chemoresistance through antagonizing miR-145-5p [97]. And it has been manifested that VEGF-C regulated by CCL2 contributes to lymphangiogenesis and LNM in bladder cancer [98]. PROX1-dependent regulation of VEGFR3 expression is conducive to lymphatic differentiation in LECs modulated by FAO [60]. A lymphatic-specific deletion of CPT1A results in defective lymphatic vessels formation and function [60], which displays restraint of forming LNM. Upregulation of epidermal growth factor in melanoma cells can mediate lymph node micrometastasis via lymphangiogenesis [99]. Erlotinib, an epidermal growth factor receptor inhibitor, is used for cancer therapy indicating FAO diminished [100]. In addition, the CCR7 signaling and CCL1/CCR8 are two of signaling pathways for breast cancer, melanoma and esophageal cancers to colonize in lymph nodes [101]. However, there is no publications on the role of CCR7 signaling and CCL1/CCR8 signaling in FAO.

### Vascular remodeling

**Fig. 3:**
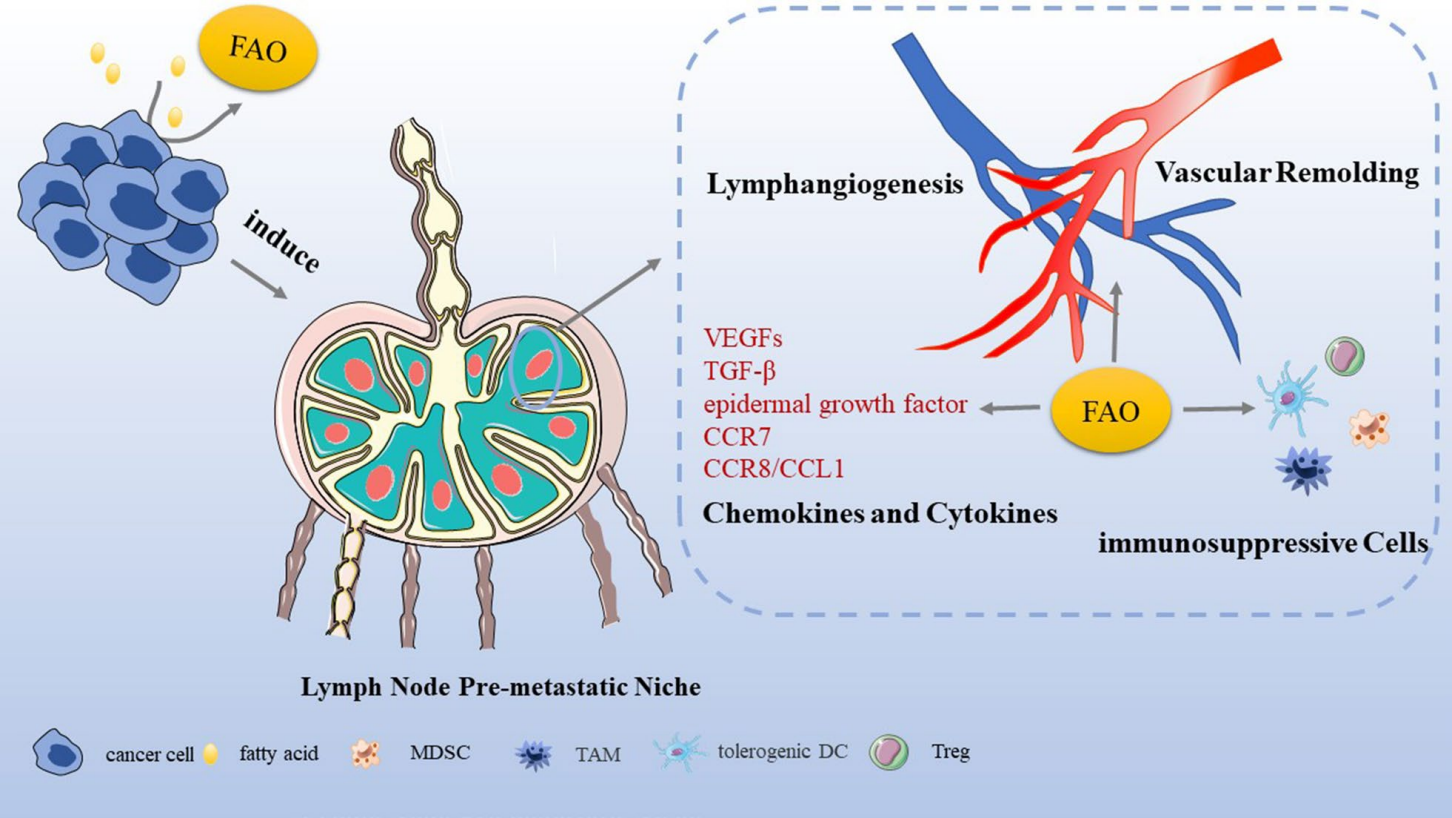
Induction and formation of LNM by FAO. FAO is implicated in multiple aspects of tumorigenesis including cancer cell growth, survival, drug resistance, and metastasis. Once migration into circulation system, the first challenge for the survival of cancer cells is to restrain apoptosis and cancer cells utilize fatty acids to fuel the growth. Then, FAO in tumor-associated immune cells and endothelial cells is also reprogrammed to contribute to immune suppression and the formation of tumor-promoting microenvironment. Lymph nodes are induced by cancer cells to establish pre-metastatic niches where FAO is involved in lymphangiogenesis, the recruitment of immunosuppressive cells, the up-regulation of chemokines and cytokines as well as vascular remodeling, indicating that FAO may play roles in formation of lymph node pre-metastatic niches. Once cancer cells colonize within lymph nodes, LNM is formed successfully

Vascular remodeling means tumor cell-induced microvascular growth and the establishment of blood circulation in TME. The pre-metastatic niches may increase angiogenesis and vascular permeability to promote metastasis. Extensive pre-metastatic vascular remodeling in lymph nodes draining nasopharyngeal and breast tumors has been reported [102, 103]. IL-17A stimulates angiogenesis by enhancing FAO through AMP-activated protein kinase (AMPK) signaling pathway [104]. Antiangiogenic drug-induced tumor hypoxia increases uptake of free fatty acids and initiates FAO metabolic reprogramming stimulating cancer cell proliferation [105]. Inhibition of CPT1significantly lowers free fatty acid-induced cell proliferation [105] (Fig. 3).

## FAO involved in maturation of lymph node niche

CTCs extravasated from vasculature and lymph vessels may enter the primary tumor-educated lymph node pre-metastatic niche, resulting in initiation of metastasis and attracting tumor cells colonization into the niche. Elevated HIF-1 promotes extravasation and chemotaxis of tumor cells by mediating hypoxia-induced proangiogenic factors such as VEGF and platelet-derived growth factor (PDGF) [106]. Evidence demonstrates that HIF-1-mediated suppression of FAO is beneficial for cancer progression via reducing ROS production, enhancing glycolysis and activating pro-survival cancer signaling in liver cancer [107]. Once DTCs successfully survive in the lymph nodes, a series of changes in the lymph node microenvironment–lymphangiogenesis [108], increased expression of immunosuppressive cytokines [109] and augment of immunosuppressive cells will be further induced, which means lymph node pre-metastatic niches turn into maturation–metastasis formation. CTCs originated from primary tumor may extravasate from the circulation where most of CTCs are killed into lymph node pre-metastatic niches where they may develop resistance to most of treatments and stay quiescent until factors promote their growth into a metastatic lesion [110] which is called as micrometastasis. Then, this niche can host more migrated tumor cells and directly promote metastatic tumor cells to grow, expand, and progress at the niche, leading to macrometastasis [111].

## FAO leading to resistance of radiotherapy and chemotherapy

The omentum is rich in lipid where adipocytes provide fatty acids by FABP4 transportation to oxidize in order to support ovarian cancer metastasis, so identifying lipid metabolism and transport as new targets for the treatment of cancer where adipocytes are a major component of the microenvironment [112]. Similarly, lymph node is also the lipid-rich microenvironment, so targeting FAO may be the promising therapy for cancers to avoid LNM. Now there are some medicines in clinical application which can target FAO to ameliorate status of cancer therapy (Table 1). At present, postoperative chemoradiotherapy for cancer patients with LNM is the routine treatment clinically. Resistance to radiotherapy and chemotherapy poses a major challenge in cancer metastasis treatment. Accumulating studies showed that FAO activation is an important mechanism employed by cancer cells to develop drug resistance. For example, several up-to-date studies demonstrated that relative inhibitors can regulate FAO of tumor cells to inhibit metastasis and improve the sensitivity of chemotherapy (CT) and radiotherapy (RT) in triple-negative breast cancer (TNBC) and nasopharyngeal carcinoma [113]. In TNBC, a synthesized flavonoid derivative GL-V9 exhibits a potent inhibitory effect on the anchorage independent growth in vitro and anti-metastasis effect in vivo through decreasing G6PD and increasing p-ACC, that is, the level of glycolysis is suppressed, whereas FAO is highly enhanced [38]. Blocking FAO via FAO inhibitor or by CRISPR-mediated CPT1A/CPT2 gene deficiency inhibited radiation-induced ERK activation and aggressive growth and radioresistance of radioresistant breast cancer cells and radiation-derived breast cancer stem cells [114]. Upregulation of CPT1A promotes radiation resistance of nasopharyngeal carcinoma [115]. Targeting PPAR coactivator‐1α (PGC1α), an important transcriptional co‐activator in control of fatty acid metabolism [113] could sensitize nasopharyngeal carcinoma cells to radiotherapy, and targeting FAO in nasopharyngeal carcinoma with high expression of PGC1α might improve the therapeutic efficacy of radiotherapy [113]. Etomoxir, an irreversible inhibitor of CPT1, is used to block FAO, markedly improving the therapeutic effect of mice with lymph node metastatic melanoma [12] and increasing sensitivity of human metastatic breast carcinoma, colon carcinoma, leukemia and hepatocellular carcinoma to chemotherapy [116]. FAO inhibitor attenuates mesenchymal stem cell-induced FOLFOX regiment resistance in vivo [97]. These results indicate that FAO inhibitors in combination with chemotherapeutic drugs present as a promising strategy to overcome chemoresistance [97].

**Table 1:** Overview of FAO roles in cancer therapy

FAO targeting drug	Clinical application	Cancer type	Mechanism of action	References
Metformin	Medicine for type 2 diabetes	Breast cancer	Inhibiting FAO at clinical doses	[117]
Etomoxir	Retired from phase II clinical trial for diabetes and heart failures	Bladder cancer	Suppressing tumor progression and inducing cell cycle arrest via PPARγ-mediated pathway	[118]
		Malignant glioma	Targeting FAO to reduce energy production and cellular proliferation in glioma cells	[119]
		Leukemia	Decreasing leukemia cells proliferation and sensitizing leukemia cells to apoptosis induced by ABT-737 and Nutlin 3a	[120]
		Peritoneal metastatic colorectal cancer	Inhibiting cancer-associated fibroblast FAO to decline colorectal cancer metastasis	[121]
		Epithelial-derived high-grade serous ovarian cancer	Targeting FAO to promote anoikis and inhibit ovarian cancer progression	[37]
		Nasopharyngeal carcinoma	Targeting FAO to sensitize cancer to radiation therapy	[115]
Ranolazine	Medicine for angina pectoris in Europe and United States	Leukemia	Decreasing leukemia cells proliferation and sensitizing leukemia cells to apoptosis induced by ABT-737 and Nutlin 3a	[120]
Perhexiline	Medicine for angina pectoris in Australia and Asia	Leukemia	Targeting CPT to eliminate chronic lymphocytic leukemia cells in stromal microenvironment	[122]

## Conclusions

Recent progress highlights that FAO can act not only as a great force to fuel tumor growth under metabolic stress, but as an accelerator to help CTCs settle in lymph nodes, and discusses biological mechanisms of FAO in the formation of LNM. However, we still lack a systematic framework of the whole process of FAO driving CTCs to encroach on lymph nodes. Theoretically, these following unresolved issues seem worthy of further exploration:

### FAO and CTCs invasion

The roles of FAO in EMT, a priming process leading to metastasis of epithelium‐derived carcinomas has been shown. Then, CTCs will face a series of challenges during the process, including attacked by immune cells, more energy supply and metabolic toxic substance. Therefore, FAO might be a preferred fuel choice for CTCs to overcome deficiency of energy undergoing metastatic progression. Although CTCs prefer to settle in the lipid-rich environment, the relationship between CTCs and FAO will be needed to explain.

### FAO and lymph node pre-metastatic niche

Formation of lymph node pre-metastatic niches can be perceived as a complex process including lymphangiogenesis, the recruitment of immunosuppressive cells, the up-regulation of chemokines and cytokines as well as vascular remodeling, all of which are dispensable and work together. Most of the present data shows that FAO can regulate any component of lymph node pre-metastatic niches. However, what has been stated above is circumstantial evidence. Therefore, direct evidence is required to elucidate whether FAO is involved in formation of lymph node pre-metastatic niches. And how much role does FAO play in pre-metastatic niche and its metastasis promoting function?

### FAO and establishment of immunosuppression

The activation of FAO pathway in immunosuppressive cells may be potential mechanism to induce immune tolerance and hence promote metastasis formation. However, what are the molecular mechanisms underlying the ability of immunosuppressive cells to rapidly switch their metabolic and functional profile?

### Therapeutic implications and FAO

It has been realized over the past few decades that FAO fortifying the CT and RT resistance attenuates effectiveness of cancer therapy, which worsens conditions of cancer patients, so it is likely that agents that target FAO will need to be combined with chemotherapies or with radiotherapies. Clearly, further knowledge of the dependence of cancer cells in LNM on FAO will be important to refine rational approaches to combination therapies.

## Data Availability

All available.

## Abbreviations

FAO: Fatty acid oxidation
TME: Tumor microenvironment
LNM: Lymph node metastasis
FATP: Fatty acid transport protein
FABP: Fatty acid binding protein
CPT1: Carnitine palmitoyltransferase 1
CPT2: Carnitine palmitoyltransferase 2
PPARs: Peroxisome proliferator-activated receptors
ACC: Acetyl-CoA carboxylase
AMPKs: AMP-activated protein kinases
CTCs: Circulating tumor cells
EMT: Epithelial–mesenchymal transition
YAP: YES-associated protein
TAZ: Transcriptional coactivator with PDZ-binding motif
ACADL: Long-chain acyl-CoA dehydrogenase
DTCs: Disseminated tumor cells
TAMs: Tumor-associated macrophages
Tregs: Regulatory CD4^+^ T cells
MDSCs: Myeloid-derived suppressor cells
DCs: Dendritic cells
HIF-1α: Hypoxia-inducible factor 1α
VEGFR3: Vascular endothelial growth factor receptor 3
PDGF: Platelet-derived growth factor
CT: Chemotherapy
RT: Radiotherapy
TNBC: Triple-negative breast cancer
PGC1α: PPAR coactivator‐1α
